# Effectiveness of Telemedicine in Inflammatory Bowel Disease in Russia: TIGE-Rus (Telemonitoring for IBD Goodness Examination in Russia) Study Protocol of a Randomized Controlled Trial

**DOI:** 10.3390/jcm13247734

**Published:** 2024-12-18

**Authors:** Dina A. Akhmedzyanova, Yuliya F. Shumskaya, Yuriy A. Vasilev, Anton V. Vladzymyrskyy, Olga V. Omelyanskaya, Yulya A. Alymova, Marina G. Mnatsakanyan, Alexandr S. Panferov, Olga V. Taschyan, Irina V. Kuprina, Marta V. Yurazh, Artur S. Eloev, Roman V. Reshetnikov

**Affiliations:** 1 Research and Practical Clinical Center for Diagnostics and Telemedicine Technologies of the Moscow Health Care Department, Moscow 127051, Russia; shumskayayf@zdrav.mos.ru (Y.F.S.); npcmr@zdrav.mos.ru (Y.A.V.); vladzimirskijav@zdrav.mos.ru (A.V.V.); omelyanskayaov@zdrav.mos.ru (O.V.O.); alymovaya@zdrav.mos.ru (Y.A.A.); reshetnikovrv1@zdrav.mos.ru (R.V.R.); 2 Gastroenterology Department, The First Sechenov Moscow State Medical University (Sechenov University), Moscow 119991, Russia; mnatsakanyanmg@gmail.com (M.G.M.); panferov_a_s@staff.sechenov.ru (A.S.P.); olgatash1@rambler.ru (O.V.T.); kuprina_i_v@staff.sechenov.ru (I.V.K.); yurazh_m_v@staff.sechenov.ru (M.V.Y.); mr.eloev@bk.ru (A.S.E.)

**Keywords:** inflammatory bowel disease, ulcerative colitis, Crohn’s disease, mHealth, telemonitoring

## Abstract

**Background**: Inflammatory bowel diseases (IBD), associated with a significant burden on patients’ lives, are becoming increasingly common. Patients with IBD need continuous treatment and lifelong monitoring, which could be achieved by telemonitoring. Telemonitoring has been shown to be effective in improving outcomes for patients with IBD, and can provide a more convenient and accessible way for patients to receive care. However, the certainty of evidence remains low. This article outlines the methodology of a randomized control study that aims to assess the efficacy of telemonitoring compared to face-to-face follow-up for patients with IBD in Russia, hypothesizing that the implementation of telemonitoring will lead to improvement in clinical, social, and organizational areas. **Methods:** The TIGE-Rus study is a randomized controlled trial. The study consists of three stages, including selection of patients and random assignment into two groups with a ratio of 1:1, follow-up care using telemonitoring or face-to-face appointments, and evaluation and comparison of follow-up efficacy in both groups. In the first stage, all patients will undergo laboratory tests and instrumental examinations, and fill out questionnaires to measure disease activity, quality of life, medication adherence, psychological well-being, and satisfaction with medical care. In the second stage, the control group will receive standard care while the telemonitoring group will have access to a web platform where they can report their clinical activity, fill out questionnaires, and have online consultations with gastroenterologists. The gastroenterologists will also make monthly phone calls to each patient in the telemonitoring group to monitor their progress. In the third stage of the study, both the telemonitoring group and the control group will be re-hospitalized after six months of monitoring. IBD activity will be evaluated through laboratory and instrumental examinations. Additionally, all the participants will complete questionnaires to assess the disease activity, medication adherence, quality of life, psychological well-being, and satisfaction with medical care in both groups. **Conclusions**: The trial will explore whether telemonitoring is effective in improving clinical, social, and organizational aspects in the management of patients with IBD in the setting of the Russian healthcare system.

## 1. Introduction

Inflammatory bowel diseases (IBD), such as Crohn’s disease (CD) and ulcerative colitis (UC), are chronic conditions characterized by recurrent inflammation of various parts of the gastrointestinal tract. The increasing prevalence and incidence of IBD [1,2], combined with the nature of the disease course, creates a significant medical, social, and financial burden [3]. Even after achieving remission, patients may still experience symptoms such as stool disorders, abdominal pain, and weakness, leading to reduced ability to work and socialize, deterioration of quality of life, and psychological distress [4].

The Selecting Therapeutic Targets in Inflammatory Bowel Disease (STRIDE II) consensus in 2021 recommended that the endpoint for treatment should not only be clinical remission but also improvement in health-related quality of life (QoL), which could be achieved by continuous lifelong follow-up [5]. However, longitudinal face-to-face follow-up is resource-intensive [3], encouraging the development and implementation of innovative solutions and online tools, including telemedicine technologies (TMT) [6], large language models [7], artificial intelligence, and machine learning [8].

Telemonitoring has been shown to be effective in improving outcomes for patients with IBD [9]. Additionally, telemonitoring can provide a more convenient and accessible way for patients to receive care, as they can communicate with their healthcare provider from the comfort of their own homes. According to a systematic review by Al Khoury et al., IBD patients have a positive attitude towards the use of TMT and expect it to be included into their treatment program [10]. A meta-analysis by Pang et al. demonstrated that TMT significantly improved the QoL associated with IBD (*p* = 0.002) [11]. According to Cross et al., IBD patients in the telemonitoring group had a lower risk of hospitalization in comparison with the standard-care group [12]. However, previous studies estimated ad hoc endpoints that despite their value did not provide a complex assessment of social, organizational, and clinical aspects. To address this gap, before the start of the trial we defined the list of assessed parameters by the Delphi method [13]. In our study, we consider the individual with IBD not only as a patient but also as a person and a consumer of medical services.

Our study hypothesizes that the implementation of TMT in patient monitoring will lead to improvements in three key aspects:

(i) Clinical aspects: a reduction in the number of relapses and in disease activity;
(ii) Social aspects: improvements in QoL and psychological well-being;
(iii) Organizational aspects: higher adherence to treatment and satisfaction with medical care.

Additionally, the study hypothesizes that patients’ QoL will be influenced by both the clinical course of the disease and their psychological well-being.

This article outlines the methodology of a randomized control study that aims to assess the efficacy of telemonitoring compared to face-to-face follow-up for patients with IBD in Russia.

### Objectives

The primary objective of this study is to assess the impact of telemonitoring on quality of life (QoL) in patients with inflammatory bowel disease (IBD). Secondary objectives include evaluating disease activity, the incidence of IBD relapses, and the rate of leukopenia in patients receiving immunomodulatory treatments (e.g., thiopurines, cyclosporine, tacrolimus). Additionally, the study will investigate medication adherence, psychological well-being, and patient satisfaction with medical care in the telemedicine group, compared to the face-to-face follow-up group. Finally, we aim to explore the relationship between secondary outcomes and QoL.

## 2. Materials and Methods

This trial protocol follows the SPIRIT (Standard Protocol Items: Recommendations for Interventional Trials) 2013 guidelines [14]. The trial will be conducted in compliance with The International Council for Harmonization of Technical Requirements for Pharmaceuticals for Human Use Good Clinical Practice (ICH GCP). The trial is registered on Clinicaltrials.gov in August 2023, NCT05994716.

### 2.1. Study Design

The TIGE-Rus is designed as a prospective, parallel, two-armed, randomized controlled trial with a 1:1 allocation.

**Figure 1:**
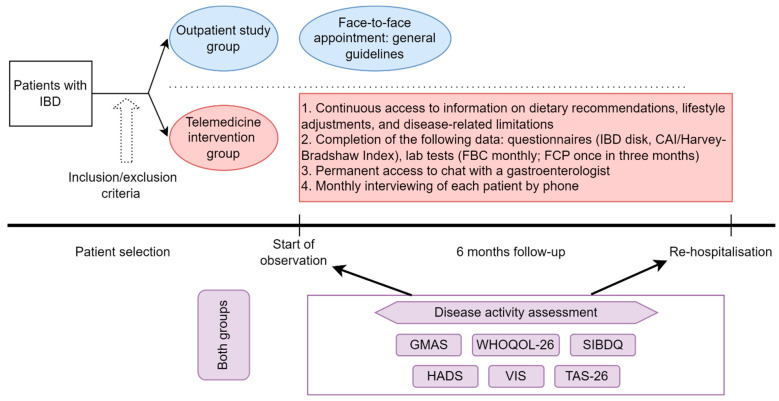
Study design.

This study will consist of three stages (Figure 1).

The first stage will be a selection of patients with IBD after treatment in the Gastroenterology Department of the Sechenov University Hospital and random assignment of participants to two groups: face-to-face outpatient observation (control group) and observation using telemedicine technologies (intervention group). For every included patient, evaluation of disease activity, QoL, medication adherence, psychological well-being, and satisfaction with medical care will be performed (Figure 1).

The second stage consists of the follow-up care. For the control group, the follow-up scheme includes face-to-face appointment and follow-up recommendations on treatment, post-discharge care plan, and diet. They will be provided with the recommendations on discharge from the hospital and then on the patient’s request. The follow-up for the intervention group consists of the following: monthly completion of questionnaires on the specialized web platform by the patient; the possibility of contacting the gastroenterologist via chat or phone call on the patient’s request; and access to educational information about IBD, psychological well-being, lifestyle, diet, sexual life, pregnancy (examples in Multimedia Appendix A), posted on the web platform. In addition, patients in the intervention group will receive a monthly phone call to address any questions or concerns they may have. During these calls, they will also be interviewed using a predefined checklist (Multimedia Appendix B).

The third stage of the study will be evaluation and comparison of follow-up efficacy in the control and intervention groups. All patients will be re-hospitalized to the Gastroenterology Department after 6 months of follow-up, where the QoL, disease activity, number of IBD relapses, frequency of leukopenia in patients receiving immunomodulators, medication adherence, psychological well-being, and satisfaction with medical care will be assessed (Figure 1).

### 2.2. Study Setting and Eligibility Criteria

The study will be conducted in the Gastroenterology Department of the Sechenov University Hospital in Moscow, Russia. It is a national center where patients from all Russian regions are treated. Thus, the study results can be extrapolated to the entire Russian population.

#### 2.2.1. Inclusion Criteria:

(1) Age ≥ 18 years old. Under Russian law, age 18 is the point at which patients transition from the pediatric to the adult population. The aim of our study is to assess the effectiveness of telemonitoring in adult patients with inflammatory bowel disease. There is no upper age limit in our study because, as long as the patient does not meet any exclusion criteria, age will not influence the study outcomes;
(2) Signed informed consent;
(3) Diagnosis: Crohn’s disease and ulcerative colitis (the diagnostic criteria for Crohn’s disease and ulcerative colitis are detailed in Multimedia Appendix C);

#### 2.2.2. Exclusion Criteria:

(1) Severe cognitive dysfunction;
(2) Severe mental illness;
(3) Oncological diseases requiring active treatment;
(4) Decompensation of a comorbid condition that has worsened to the point of posing serious health risks or complicating the assessment of the trial’s outcomes;
(5) Pregnant individuals;
(6) Participation in other clinical studies;
(7) Lack of technical skills to take part in telemedicine intervention (e.g., difficulty using a smartphone, computer, or tablet) or the absence of appropriate technology;
(8) Inability to understand written Russian.

### 2.3. Consent

Every patient with IBD will receive an information brochure. A trained researcher will introduce the TIGE-Rus details to participants and discuss the trial with them. If the patient agrees to participate, they will have to sign informed consent (Multimedia Appendix D).

Patients who will be allocated to the telemonitoring group will also receive an Addendum to the informed consent containing information on health conditions requiring emergency or urgent care (Multimedia Appendix E).

### 2.4. Randomization

Randomization will be performed in 1:1 ratio between control and experimental groups using the envelope method.

### 2.5. Trial Interventions and Participant Timeline

#### 2.5.1. Stage 1

All participants will undergo a series of laboratory tests, including a complete blood count, C-reactive protein levels, and fecal calprotectin. They will also receive instrumental evaluations such as a colonoscopy with biopsy. For patients with severe IBD or jejunoileitis, a contrast-enhanced abdominal and pelvic CT scan or magnetic resonance enterography will be conducted (Table 1).

After group assignment and signing informed consent, all participants will fill out the following questionnaires:

- Simple Clinical Colitis Activity Index (SCCAI) [15] questionnaire for patients with ulcerative colitis/Harvey-Bradshaw index (HBI) [16] questionnaire for patients with Crohn’s disease;
- Short Inflammatory Bowel Disease Questionnaire (SIBDQ) [17];
- World Health Organization’s QoL (WHOQOL-26) [18];
- General Medication Adherence Scale (GMAS) [19]
- Patient Satisfaction Questionnaire (PSQ-18) [20];
- Hospital Anxiety and Depression Scale (HADS) [21];
- Visceral Sensitivity Index (VSI) [22];
- Toronto Alexithymia Scale (TAS-26) [23].

The clinical activity of the disease will be measured by Disease Activity Index (DAI) for ulcerative colitis and Crohn’s Disease Activity Index (CDAI) for Crohn’s disease [24,25].

#### 2.5.2. Stage 2

The patients in the control group will have a face-to-face consultation with a gastroenterologist, who will offer treatment recommendations, a post-discharge care plan, and dietary advice. Standard care for the control group will follow evidence-based guidelines, with outpatient visits available upon the patient’s request [24,25].

**Figure 2:**
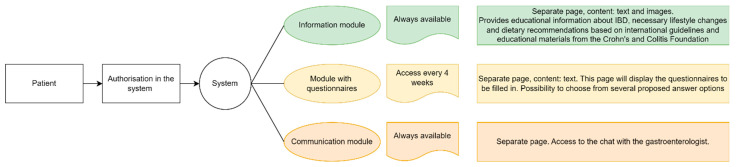
An online platform structure for patients in the intervention group.

The telemonitoring group patients will receive authorized access to the personal account on the web platform. The structure of the web platform is shown on the Figure 2. It includes educational content on IBD, necessary lifestyle adjustments, and dietary guidelines, all based on international standards and resources from the Crohn’s and Colitis Foundation.

Patients will be required to log in to the web platform at least once per month. Upon logging in, they will need to provide the following information: (1) SCCAI for ulcerative colitis and the Harvey-Bradshaw index for Crohn’s disease to assess disease activity during monitoring; (2) the IBD disk questionnaire to track disease progression; and (3) results from laboratory tests (complete blood count, C-reactive protein, and fecal calprotectin). Both healthcare professionals and patients will have access to the test results. Additionally, patients can request an online consultation with a gastroenterologist via chat or phone call. They will be advised to contact a gastroenterologist if they experience disease recurrence.

Each month, gastroenterologists will call patients in the intervention group and ask questions based on a checklist (Multimedia Appendix A). They will be trained to provide immediate assistance if there are critical deviations in health indicators from the reference values (see Table 2) or complaints suggesting the onset of an acute condition.

##### Web Platform Description

**Figure A1:**
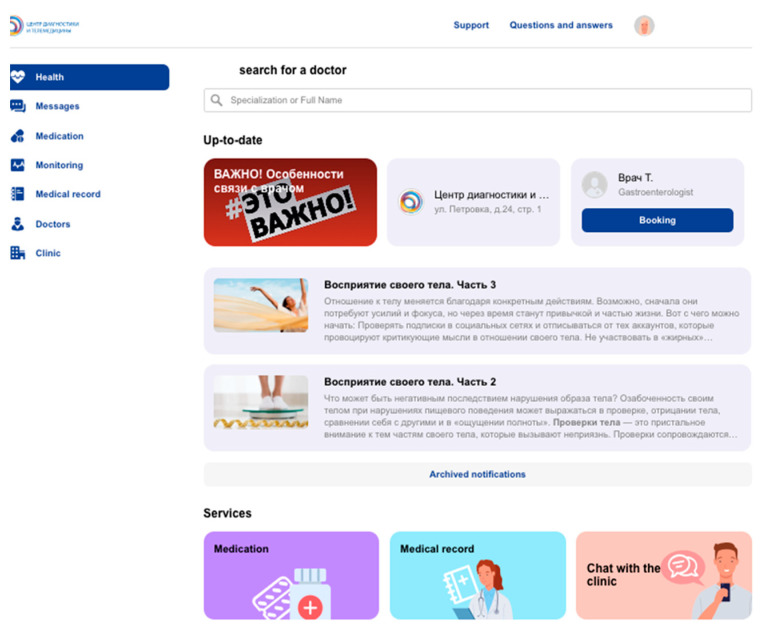
Personal patient profile, newsfeed with educational information.

**Figure A2:**
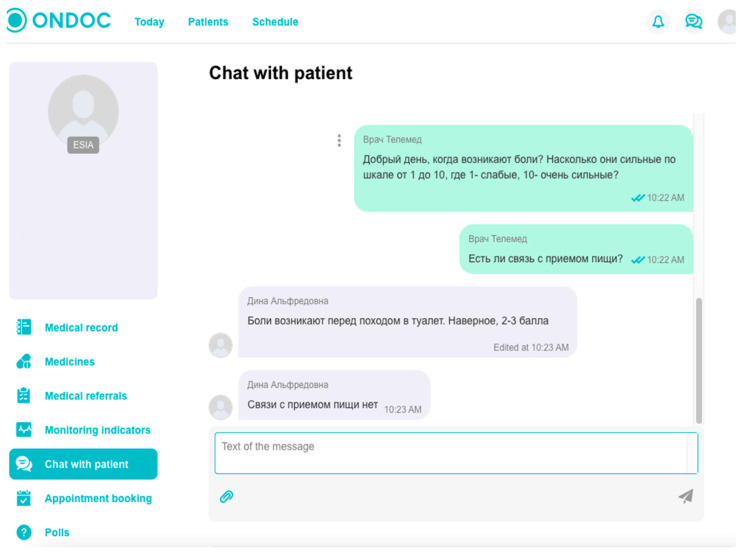
Chat with the gastroenterologist.

**Figure A3:**
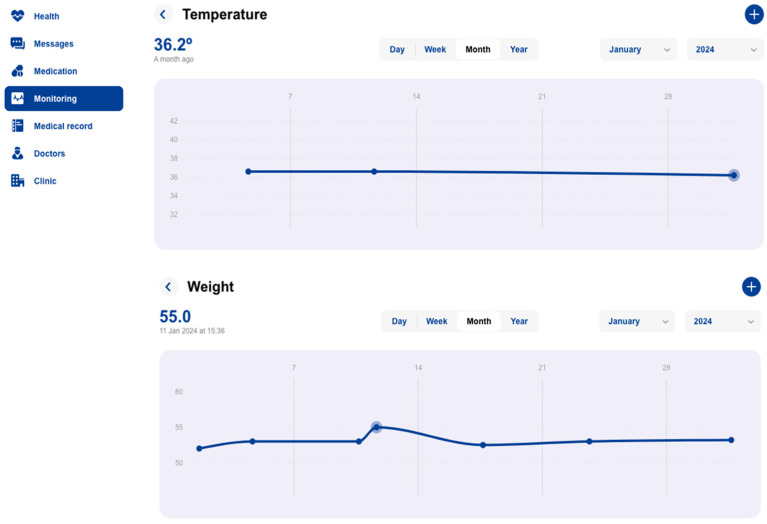
Health parameters monitoring page.

**Figure A4:**
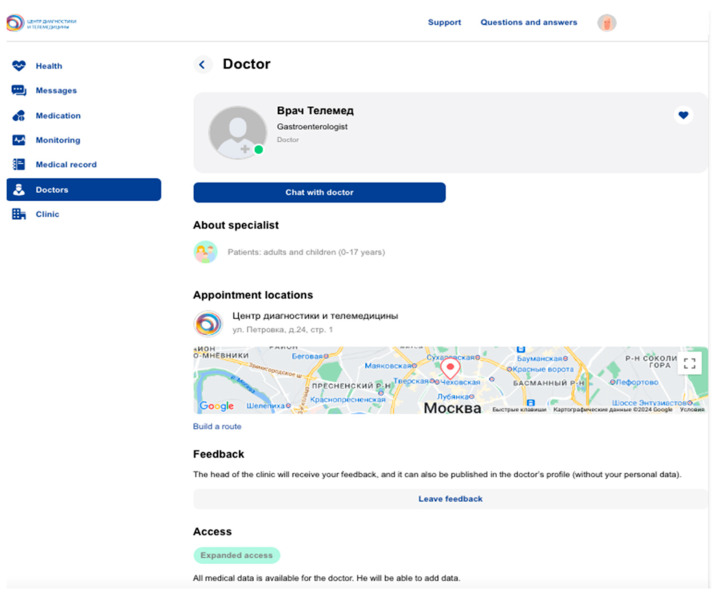
Information about the gastroenterologist.

**Figure A5:**
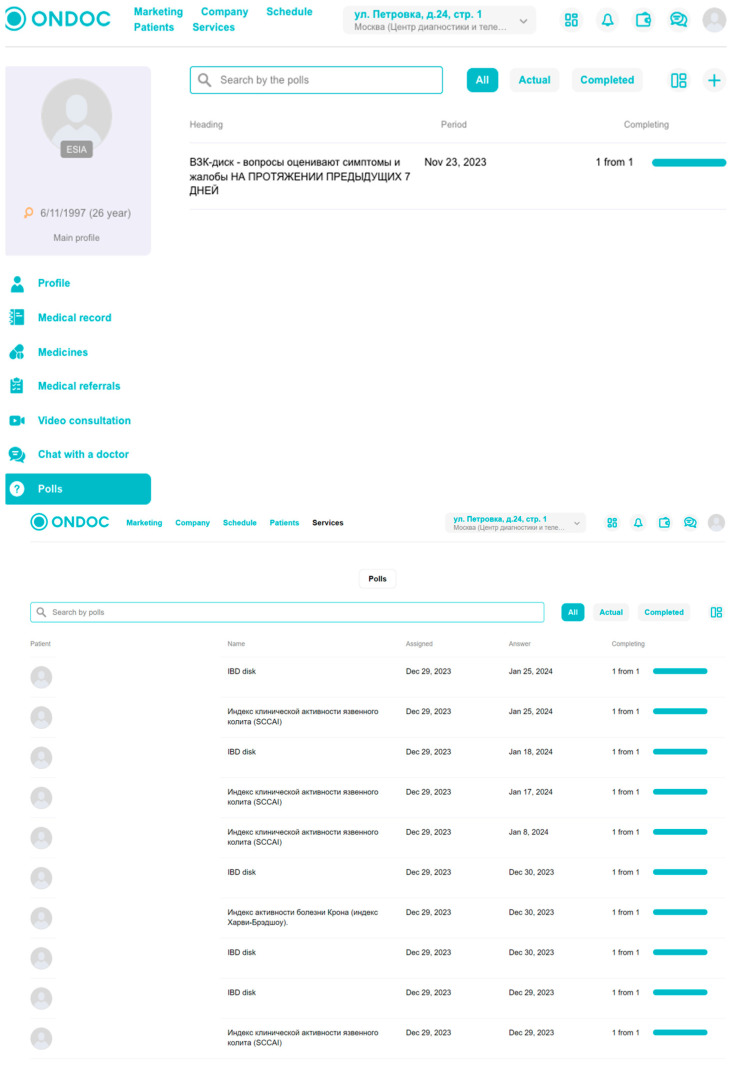
The questionnaires module.

Web platform http://ondoc.telemedai.ru/ provides access to a personal patient profile, a newsfeed with educational information (Appendix F, Figure A1), chat with the gastroenterologist (Appendix F, Figure A2), a health parameters monitoring page (Appendix F, Figure A3), information about the gastroenterologist (Appendix F, Figure A4), and the questionnaires module (Appendix F, Figure A5).

#### 2.5.3. Stage 3

After six months of monitoring, participants from both groups will be readmitted to the hospital. IBD activity will be assessed through laboratory and instrumental tests, including a complete blood count, C-reactive protein levels, fecal calprotectin, colonoscopy with biopsy, and computed tomography or MR-enterography (for patients with severe IBD or jejunoileitis). Both groups will also have to recomplete all the questionnaires to evaluate the study endpoints (see Table 1).

### 2.6. Adverse Event Reporting and Harms

An adverse event is defined as any untoward health-related occurrence in a study participant. It does not necessarily have a correlation with the allocated intervention. However, any adverse event will be recorded and reported at any study time point. Nevertheless, we developed an Addendum to the informed consent containing information on health conditions requiring emergency or urgent care for the intervention group (Multimedia Appendix D). We do not anticipate any harm related to participation in the study.

### 2.7. Outcome Measurements and Data Collection Methods

The assessment of the study outcome-related variables will take place at the baseline and at 6 months post–group assignment (Table 3).

### 2.8. Sample Size

The sample size was determined by the objective of estimating the primary outcome of the study. The standard deviation and expected difference in disease-related QoL between groups for the sample size were based on data from studies of IBD patients assessed with SIBDQ. The standard deviation was taken to be 12.52 points based on the study by Sun et al. [27]. The expected difference between the study groups was chosen to be smaller than the clinically significant change in HRQoL according to Jowett et al. [28] and taken to be 10 points. Considering a potential loss and incomplete records of 20%, a total of at least 64 patients (32 patients in the control group and 32 patients in the intervention group) should be included in the study to detect a difference between groups with a statistical power of 80% (two-sided type I error of 0.05).

### 2.9. Statistical Methods

The results will be analyzed only after the follow-up of all the included patients has been completed. The questionnaire scores will be calculated based on scoring guides from the questionnaire developers. Missing questions will be processed according to these guidelines. Patients without completed SIBDQ at any study point will be excluded from the analysis. Patients who refused to participate at any point in the study will be excluded from the analysis. We plan to use the full analysis set and the per protocol set (for patients without completed SIBDQ at any study point or who refused to participate at any point in the study).

Continuous variables will be tested for normality using the Shapiro–Wilk test and presented as mean (SD) or median (IQR), as appropriate. Categorical variables will be presented as percentages. For demographic and clinical data, descriptive statistics will be used to characterize the study population and to identify erroneous values. Additionally, missing values will be analyzed to determine the randomness of these omissions.

Hypothesis testing will be conducted for primary and secondary outcomes (Table 4). Quantitative variables will be compared using the Student’s T-test or the Mann–Whitney U test, as appropriate, and qualitative variables will be compared using the Fisher’s exact test. Univariate analysis of variance and multiple linear regression will be performed to analyze the association of secondary outcomes with the primary outcome. The Benjami and Hochberg (BH) false discovery rate (FDR) approach will be used to correct for multiple comparisons (*p* < 0.05). For the variables with FDR ≤ 10%, the term “showing a trend” will be used to avoid confusion with statistically significant variables.

All the analysis will be performed using a Python version of at least 3.7.0 or an R version of at least 4.2.0. A value of *p* < 0.05 will be considered statistically significant.

## 3. Discussion

### 3.1. Overview

In this article, we describe the key elements of the design of a randomized control study aimed to evaluate telemonitoring efficacy for IBD in Russia.

The available evidence has shown that IBD patients have a lower quality of life compared to healthy individuals [29], even during periods of remission [30]. IBD is characterized by a relapsing and remitting clinical course that requires lifelong monitoring. Telemonitoring offers a promising solution by enabling the continuous monitoring of a wide range of health-related parameters. A recent systematic review has indicated that telemonitoring improved the QoL for individuals with IBD [11]. However, the systematic review of Nguyen et al., which employed the GRADE (Grading of Recommendations, Assessment, Development, and Evaluations) approach, showed a reduction in healthcare utilization and costs, with no change in QoL, disease activity, or medical adherence (low or very low certainty of evidence) [31].

These results highlight the need for further studies to better understand the true impact of telemonitoring on IBD patients. The studies included in the reviews assessed various parameters and used different metrics, which shows the complexity of evaluating telemonitoring in IBD. To address this complexity, we decided to evaluate the impact of telemonitoring on a broad range of parameters, including clinical, social, and organizational aspects (see Table 1). Previously, we approached the issue systematically and surveyed gastroenterologists specializing in IBD treatment. Thus, we determined the parameters that should be monitored in IBD patients during telemonitoring [13]. It is important to note that this is the first trial in Russia aimed to evaluate telemonitoring efficacy in IBD.

When considering the scalability of an intervention, we can hypothesize that telemonitoring might be a suitable option for IBD patients in remote areas who do not have direct access to qualified face-to-face medical care. After the study completion, we plan to use the Intervention Scalability Assessment Tool (ISAT) [32] for the scalability assessment. In Russia, there are already examples of telemonitoring being implemented at the state level, such as the Federal project ‘Personal Medical Assistants’ https://ppma.ru/, which provides remote monitoring for patients with type 2 diabetes and arterial hypertension, funded by compulsory medical insurance.

### 3.2. Expected Findings

We anticipate that the implementation of TMT in monitoring patients with IBD will improve their QoL. This will be achieved through a reduction in overall and visceral anxiety, as well as constant, immediate access to medical care. Additionally, we expect an increase in satisfaction with medical care, improved psychological well-being, and a decrease in disease activity and relapse rate due to timely response and improved adherence to treatment.

### 3.3. Strengths

This study has been designed in close collaboration with patients to ensure that it addresses their specific needs and concerns. We discovered that there is a lack of standardized criteria for evaluating patients with IBD during monitoring except for objective markers of disease activity. We defined the list of assessed parameters by the Delphi method before the trial [13]. Another advantage of this trial is that the protocol has been developed in accordance with the SPIRIT guidelines, which will improve its transparency [14].

### 3.4. Limitations

The study has some limitations. One potential limitation is an uneven distribution of patients with UC and CD within the groups. Due to envelope randomization, there may be differences in the number of participants with UC or CD between the face-to-face and telemonitoring groups. Furthermore, the study does not intend to perform subgroup analysis based on a specific disease, such as UC or CD.

Another limitation of this study is that it is a single-center study. Different hospitals may have slightly different approaches to face-to-face management of patients. Additionally, patients in the groups may differ in the activity and severity of their IBD course, which could impact the therapy they receive during the study period.

Furthermore, the use of a website as a telemedicine intervention may also be a limitation. This approach requires patients to have certain technical equipment and computer literacy, which could reduce the number of study participants.

## 4. Conclusions

Our study aims to assess the effects of telemonitoring on patients with IBD in comparison to traditional face-to-face follow-up. Specifically, we will evaluate the impact on various aspects, such as QoL, frequency of disease relapses, medication adherence, adverse drug reaction of immunomodulators, and satisfaction with medical care.

## Figures and Tables

**Table 1:** Summarizes the trial interventions and participant timeline.

	Study Period						
	Enrolment/Baseline 0 Weeks	Follow-Up					End of the Study (6 Months)
		1 Month	2 Months	3 Months	4 Months	5 Months	
Informed consent	X						
Eligibility criteria	X						
Demographic data	X						
Treatment in the Gastroenterology Department	X						X
Adverse events		X	X	X	X	X	X
Treatment	X	X	X	X	X	X	X
Face-to-face consultation for both groups	X						X
Observation using telemedicine technologies for the intervention group (including web-platform, phone calls)		X	X	X	X	X	
Clinical parameters							
Haemoglobin concentration	X	X	X	X	X	X	X
White blood cells concentration	X	X	X	X	X	X	X
C-reactive protein concentration	X			X			X
Fecal calprotectin concentration	X			X			X
IBD disk		X	X	X	X	X	
HBI/SCCAI questionnaire	X	X	X	X	X	X	X
Disease severity according to laboratory and instrumental data	X						X
Disease severity according to CDAI/UCDAI	X						X
Social parameters							
VSI questionnaire	X						X
TAS-26 questionnaire	X						X
HADS questionnaire	X						X
SIBDQ questionnaire	X						X
WHOQOL-26 questionnaire	X						X
Organizational parameters							
PSQ-18 questionnaire	X						X
GMAS questionnaire	X						X

**Table 2:** Limit values of the laboratory tests.

Laboratory Parameter	Critical Deviation from Reference Values
Haemoglobin concentration	Lower than 110 g/L; higher than 170 g/L
White blood cells concentration	Lower than 3 × 10^9^ cells/L; higher than 11 × 10^9^ cells/L
C-reactive protein concentration	Higher than 10 mg/L
Fecal calprotectin concentration	Higher than 200 µg/g for patients included in the trial with initially normal levels. Maintaining the level of more than 800 µg/g between two time points (Table 1) for patients included in the trial with initially high levels

**Table 3:** Study outcomes and data collection methods.

Item	Definition	Data Collection Method	Measure
Primary outcome			
Health-related quality of life (HRQol) in IBD	QoL specifically associated with bowel symptoms	SIBDQ score	Min score is 10, max score is 70. <50 means poor HRQol; >50 optimal HRQol Higher score means better outcome
Secondary outcomes			
Generic QoL	Generic QoL associated with several domains of life quality (physical and mental health, social relationships, and environment)	WHOQOL-26 score	Min score is 0%, max score is 100% for each domain. Higher score means better outcome.
Clinical activity of UC	Clinical activity of UC according to DAI with questions regarding clinical symptoms and endoscopic activity	DAI	0–2—remission; 3–6—mild activity; 7–10—moderate activity; >10—severe activity;
Clinical activity of CD	Clinical activity of CD according to CDAI, which is a ‘gold standard’ for trials. CDAI consists of questions regarding symptoms, lab tests, extraintestinal complications, general well-being	CDAI	<150—remission; 150–300—mild activity; 301–450—moderate activity; >450—severe activity;
General medication adherence	Adherence to the prescribed medications, patient compliance	GMAS score	0–26—non-adherent; 27–33—adherent [26];
Rate of leukopenia in patients taking immunomodulators (thiopurines, cyclosporine, tacrolimus)	Leukopenia that is associated with the intake of immunomodulators according to full blood count during the monitoring	Electronic health record, full blood count	Number of patients taking immunomodulators with onset of leukopenia
Satisfaction	Patient satisfaction with healthcare	PSQ-18 score	Min score is 18, max score is 90. Higher score means better outcome;
Depression and anxiety	Levels of anxiety and depression in patients with chronic diseases	HADS	The HADS has two scales: for anxiety (HADS–A) and for depression (HADS–D), differentiating the two states. For each scale: 0–7—no depression or anxiety; 8–10—a doubtful case; 11–21—a definitive case;
Visceral sensitivity	Gastrointestinal (GI) symptom-specific anxiety causing bowel symptoms persistence	VSI score	0–10—no GI-specific anxiety; 11–30—moderate GI-specific anxiety; 31–75—severe GI-specific anxiety;
Alexithymia	Difficulty to perceive and express emotions	TAS-26 score	26–62—no alexithymia; 63–74—a doubtful case; 75–130—a definitive case;
Additional outcomes			
Endoscopic activity of UC	Endoscopic activity of UC assessed via colonoscopy	Mayo Endoscopic Score (MES)	0—normal or inactive disease; 1—mild disease with erythema, decreased vascular patterns and mild friability; 2—moderate disease with marked erythema, absence of vascular patterns, friability and erosions; 3—severe disease with spontaneous bleeding and ulceration
Endoscopic activity of CD	Endoscopic activity of CD assessed via colonoscopy	Simple Endoscopic Score for Crohn’s Disease (SES-CD)	0–2—remission; 3–6—mild severity; 7–15—moderate severity; >15—severe;
Histological activity of IBD	Histological activity of IBD assessed via biopsy	Binary scale	No signs of inflammation in the histological material; Presence of signs of inflammation in the histological material
Laboratory tests	White blood cells concentration; Haemoglobin concentration; C-reactive protein concentration; Fecal calprotectin concentration;	Blood test and stool test	Normal values: 4–11 × 10^9^ cells/L; 120–170 g/L; 0–5 mg/L; <200 µg/g for patients with initially normal levels. <800 µg/g in both time-points for patients included in the trial with initially high levels
General medication adherence differences	Adherence to the prescribed medications, interpreted in 5 levels of adherence	GMAS score	0–10—poor adherence; 11–16—low adherence; 17–26—partial adherence; 27–29—good adherence; 30–33—high adherence;
Rate of non-scheduled medical encounters *	Unplanned visits to the Physician for IBD symptoms	As recorded in electronic health record	Number of non-scheduled visits
Rate of surgical interventions *	Surgical interventions for IBD complications	As recorded in electronic health record	Number of surgical interventions
Rate of hospitalizations *	Unplanned hospital admissions for any reason	As recorded in electronic health record	Number of unplanned hospitalizations with specification of the reason

* we assume that telemonitoring will lead to decrease in these parameters.

**Table 4:** Hypotheses proposed according to the primary and secondary study aims.

*Hypothesis*	**H_0_**	**H_a_**	
	6 months after the start of observation, there is no difference between the groups on the evaluated parameter	6 months after the start of observation, the values of the assessed parameter are higher in the telemonitoring group	6 months after the start of observation, the values of the estimated parameter are lower in the telemonitoring group
Outcomes	All primary and secondary outcomes	HRQol Generic QoL General medication adherence Satisfaction with medical care	Clinical activity of UC/CD Rate of leukopenia in patients taking immunomodulators (thiopurines, cyclosporine, tacrolimus) Depression and anxiety Visceral sensitivity Alexithymia

H_0_—null hypothesis; Ha—alternative hypothesis.

## Data Availability

The datasets utilized and examined in this study can be obtained from the principal investigator (Akhmedzyanova Dina) upon reasonable request.

## Appendix A

**Table A1:** Educational information (Translated from Russian).

Topic	Example of Information
General information about IBD	Inflammatory bowel disease (IBD) is a term that covers diseases causing chronic inflammation of the gastrointestinal (GI) tract. The most common forms of IBD are ulcerative colitis (UC) and Crohn’s disease (CD). Both CD and UC have similar symptoms; however, they are different disorders and affect different parts of the GI tract. The causes of IBD have not been studied enough. A combination of genetic factors (inherited) and environmental factors (acquired—a contact with animals, eating habits, etc.) is assumed. Normally, the immune system protects the body. However, in people with UC, the immune system may mistakenly take native microflora (bacteria that are normally in the intestines helping us absorb vitamins, food and other ordinary substances) for foreign agents. The body begins to attack them by sending leucocytes into the gut lining, where they cause inflammation and ulceration of the wall. The most common symptoms of CD are abdominal pain (often in the right lower abdomen) and diarrhea. There may also be rectal bleeding, decreased appetite, weight loss, and fever. The most common symptoms of UC are abdominal pain, diarrhea, blood in the stool and false urge to defecate. IBD symptoms most often tend to go through periods when they are more severe (relapse) and periods when they are much less pronounced or not present at all (remission). Both diseases are more common in Western countries; however, in recent years, increase in incidence in Russia, Asian countries, and Latin America has been noticed. For example, in North America, about 0.3% of the population has IBD, which means that approximately 2.2 million people have CD or UC. The path for a patient with IBD to remission can be long. From the onset of the disease to the moment of diagnosis, a patient usually visits many doctors, undergoes a lot of tests, examinations, and misdiagnoses. IBD is a difficult disease not only for patients, but for healthcare providers as well. Sometimes, patients feel that people around them treat them differently because of their disease. This leads to stress and attempts to isolate themselves from others. In fact, it is important to learn how to tell your loved ones and friends about your illness, how you cope with it, what difficulties you experience, and how you live a fulfilling life. After all, close people can provide invaluable support and help overcome many difficulties. Clinical symptoms of IBD can be painful, embarrassing, and debilitating. They interfere with your job, school, relationships, travels, and physical and emotional well-being. They can seriously affect a person’s quality of life, causing stress, anxiety, and depression. In this case, you need to seek help from a mental health specialist and a psychological support. You cannot suffer in silence!
Psychological well-being in IBD	What is body image? Body image is our perceptions about our own body. It is not just a picture of how we look, but a set of components: What we think about our body What we feel about our body How we act in relation to it How we represent it in space How well we are able to recognize body signals Body image may not reflect how a person actually looks. In addition, ideas about your body may change throughout your life depending on what you are going through or doing. It is hardly possible to unequivocally answer the question of what exactly influences a perception of the particular person’s body image. However, several main factors can be identified: interpersonal experience, culture, physical characteristics and their changes, and individual peculiarities. · The attitude towards the body is formed from early childhood from the experience interacting with parents and other close people. The following factors influence it: how it was customary for the family to treat your own body and the bodies of others, how physiological manifestations of the child’s body and the features of his/her appearance were perceived by the close people. · Attitudes towards the body are also shaped by what is considered attractive in society. · Each person is born with specific features of appearance, and later it undergoes various changes throughout the life. Puberty, pregnancy, heredity, injuries and illnesses, age—all of these can increase a person’s concern about his/her appearance. All of the above factors affect people in different ways. Some people manage not to be affected by this experience, while for someoneelse it turns out to be difficult to cope with its consequences. This is due to individual personality traits. For example: · Low self-esteem can become a basis for the formation of a negative body image, when a sense of inner inferiority extends to the perception of one’s appearance. · A sense of insecurity in relationships can make a person feel that something is wrong with him/her, which, in turn, can contribute to a negative attitude towards one’s own body. · Perfectionism influences the development of tension regarding one’s body—in this case, a person needs to look impeccable for other people in his actions and appearance. One way or another, it is important to everyone how they look. At the same time, there is a difference between when a person “just doesn’t like something” and when dissatisfaction with appearance acquires super-valuable significance. In the second case, it becomes a problem and seriously affects quality of life, and may be a sign of a mental disorder—dysmorphophobia or an eating disorder (ED). Body dysmorphic disorder is the obsessive preoccupation with one or more perceived physical defects. Usually these defects are invisible or slightly noticeable to others; however, they are much more significant for the person. A key pathology of an ED is the over-value of one’s own body and a control over it. A negative body image does not lead to the eating disorder in every case; however, it contributes to developing and continuing this disorder in individuals predisposed to it. Normally, people’s self-esteem is based on their achievements in various areas of their lives. People with an eating disorder base their self-esteem entirely or mainly on judgments about their weight and body shape and their ability to control them. This is why body image disorder therapy plays a significant role in recovery from an eating disorder.
Diet in IBD	What can you eat during a relapse? Do not follow restrictive diets. Resist the urge to follow diets recommended to you by your friends, relatives, or people on the internet. Like medications, restrictive diets have potential side effects. They include nutritional deficiencies, unplanned weight loss, and the onset and/or progression of an eating disorder. All of these can negatively affect the disease outcome. Only your doctor or dietitian can prescribe you a correct diet. 2. Increase the amount of protein in your diet. During a relapse of IBD, the protein need is increased, so it is helpful to eat high-protein foods throughout the day. However, you should not exceed 80–100 g of protein a day. The following products are recommended: chicken, tofu, fish, turkey, eggs, cottage cheese, yogurt, beans, chia seeds, and nut butters. 3. Increase the amount of consumed liquid. If you have frequent loose stools or constipation, you need to increase the amount of consumed liquid, such as water, weak herbal teas, compotes, and hydrating solutions (for example, Rehydron). 4. High nutrient dense meals/snacks. If you have a decreased appetite, you have recently lost weight unintentionally, or you have diarrhea, then frequent eating of small portions of food can help you. You can also supplement your diet with enteral nutrition.

## Appendix B

**Table A2:** Phone call checklist (Translated from Russian).

Item	Question	Clarifying Additional Question (If Necessary)	Answer Options			
Bowel frequency	How many times a day do you have bowel movements?	How many times a day do you have bowel movements during a disease remission?	The usual	1–2 times a day more than usual	3–4 times a day more than usual	5 times a day more than usual
Blood in the stool	Is there an admixture of blood in the stool?	Is there blood in the stool itself, at the end of defecation, or on a toilet paper?	Not	Blood streaks	Visible blood	Mostly blood

**Question**	**Yes**	**No**
Do you have constipation? (a need to strain during defecation, hard or sheep-like stools, a feeling of incomplete evacuation after defecation)		
Do you have painful urges to defecate?		
Have you lost more than 3 kg without any obvious reasons?		
Have you taken any antibiotics in the last month?		
Have you taken NSAIDs (non-steroidal anti-inflammatory drugs, painkillers) in the last month?		
Do you have joint pain?		
Have you had fever above 38 °C unrelated to a cold during the last month?		
**Question**	**Yes**	**No**
Do you remember to take all your medications?		
Are you sometimes careless about the time of taking your medications?		
Do you skip taking medications when you feel well?		
If you feel unwell after taking a medicine, do you skip the next dose?		

## Appendix C. IBD Diagnostic Criteria According to Current Clinical Guidelines in Russia

### Appendix C.1. Diagnostic Criteria for Crohn’s Disease (CD) [25]

The Lennard-Jones criteria for a reliable diagnosis of CD include the following seven key features:

- Localization anywhere in the gastrointestinal (GI) tract, from the oral cavity to the anal canal, including chronic granulomatous lesions of the mucosa in the lips or cheeks, pyloroduodenal lesions, small intestine lesions, and chronic perianal lesions.
- Intermittent nature of the lesions.
- A transmural character of the lesions, which may present as fissure ulcers, abscesses, or fistulas.
- The presence of fibrosis, such as strictures.
- Lymphoid tissue findings (histology) that may include aphthoid ulcers or transmural lymphoid clusters.
- Mucin content (histology) showing normal levels in areas of active inflammation of the colonic mucosa.
- The presence of epithelioid granulomas.

A diagnosis of CD is considered reliable if three or more of these signs are present or if a granuloma is found in conjunction with any other sign.

The diagnosis must be confirmed using endoscopic and morphological methods and/or endoscopic and medical imaging techniques.

Endoscopic criteria for diagnosing CD include the presence of regional (intermittent) mucosal lesions, the ‘cobblestone’ appearance (characterized by deep longitudinal ulcers combined with transversely oriented ulcers and areas of edematous, hyperemic mucosa), linear ulcers (fissure ulcers), aphthae, and, in some cases, strictures and fistula openings.

Radiological findings associated with CD may include regional, intermittent lesions, strictures, cobblestone patterns, fistulas, and intra-abdominal or interintestinal abscesses.

Morphological features of CD include:

- Deep, slit-like ulcers that penetrate the submucosa or muscle layer.
- Epithelioid granulomas, which are clusters of epithelioid histiocytes without necrotic foci or giant cells. These are typically found in the wall of the resected area and are present in only 15–36% of cases in mucosal biopsies.
- Focal (discrete) lymphoplasmacytic infiltration of the intrinsic lamina of the mucosa.
- Transmural inflammatory infiltration with lymphoid hyperplasia affecting all layers of the intestinal wall.
- Lesions in the ileum characterized by structural changes in the villi, mucoid or pseudopyloric crypt metaplasia, and chronic active inflammation.
- Intermittent lesions, which involve the alternation of affected and healthy segments of the intestine when examining the resected portion.

### Appendix C.2. Diagnostic Criteria for Ulcerative Colitis (UC) [24]

Criteria for establishing a diagnosis based on pathognomonic findings include:

- Anamnesis (medical history);
- Physical examination;
- Laboratory tests;
- Instrumental examinations.

There are no definitive diagnostic criteria for ulcerative colitis (UC). The diagnosis is established through a combination of the patient’s history, clinical presentation, and characteristic endoscopic and histological findings.

Endoscopic examination of the colon is the primary method for diagnosing UC, although there are no specific endoscopic signs unique to the condition. The most characteristic features include diffuse inflammation confined to the mucosa, starting in the rectum and extending proximally, with a well-defined border of inflammation. The endoscopic activity of UC is best indicated by contact bleeding (the discharge of blood upon contact with the endoscope), a lack of vascularity, and the presence of erosions and ulcerations.

Microscopic signs of UC include crypt deformation, characterized by branching, multidirectional crypts of varying diameters, decreased crypt density, ‘crypt shortening’, and crypts that do not reach the underlying muscularis mucosa. Biopsies may reveal an ‘uneven’ surface of the mucosa, a reduced number of goblet cells, basal plasmacytosis, and infiltration of the lamina propria by mononuclear cells, along with a mixture of segmented neutrophils and eosinophils. Additionally, crypt abscesses and basal lymphoid aggregates may be present. Typically, the degree of inflammatory infiltration diminishes with increasing distance from the rectum.

## Appendix D. Informed Consent (Translated from Russian)

### Patient Information

Dear patient!

You are invited to participate in a study as a part of the research “Effectiveness of telemedicine technologies in monitoring patients with inflammatory bowel diseases”.

Please read this document carefully; it contains information about the study and possible risks. You can discuss all your questions with a research physician and, if you wish, with the people you trust. Once you have read this document and decided to participate in the study, you will need to sign and date two copies of the informed consent form. You will keep a signed and dated copy of the information for a patient along with the informed consent form.

Participation in the study is voluntary. If you refuse, or having signed a consent change your decision at any time during the study without explaining the reasons, it will not affect the quality of the medical care provided to you.

You are invited to participate in this study because periodic monitoring of your condition is required to minimize the risk of a worsening of your disease. Our study compares telemedicine follow-ups with office visits.

A purpose of the study is to determine whether a provision of medical care using telemedicine technologies is effective compared with conventional face-to-face observation in patients with inflammatory bowel diseases (ulcerative colitis, Crohn’s disease).

Sixty-four people are planned to participate in the study. Patients will be randomly assigned to two groups. The first group will be monitored on the outpatient basis according to the plan indicated by the attending physician in the discharge summary. Participants of the second group will be granted access to a website that will provide information about the disease, dietary recommendations, and rules of conduct. Also, the telemonitoring group will need to fill out a disease activity checklist and enter test results once a month. Patients in the telemedicine group will have the opportunity to chat with a gastroenterologist about any issues related to the disease. In addition, participants in the telemonitoring group will receive a phone call to assess their condition once a month.

On the day of your discharge from the hospital, we will ask you to complete anonymous questionnaires to achieve the following goals:

1. Clarification of your health status—the CAI questionnaire for patients with ulcerative colitis and the Harvey-Bradshaw Index questionnaire for patients with Crohn’s disease.
2. Assessment of your Quality of life—a questionnaire developed by WHO (WHOQOL-26), as well as a special quality questionnaire for patients with IBD (SIBDQ).
3. Assessment of your psychological condition—the Hospital Anxiety and Depression Scale (HADS), the Toronto Alexithymia Scale (TAS-26), which reflects a risk factor for developing psychosomatic diseases, as well as a special gastroenterological questionnaire the Visceral Sensitivity Index (VSI) to determine how well and clearly you feel the signals from your gastrointestinal tract.
4. Evaluation of our work—the Patient Satisfaction Questionnaire (PSQ-18).

Completing the questionnaires takes about 10–15 min.

After 6 months, we will offer patients a re-hospitalization as part of the disease activity assessment, where we will again ask you to complete the above questionnaires.

The duration of the participation in the study is 6 months.

Possible benefits for the patient from participating in the study are an improved quality of life, a full control over IBD, and a contribution to the development of fundamental and practical medicine.

Possible or additional risks and inconveniences associated with participation in the study are a need to spend about 5 min of personal time once a month to fill out a checklist (in the case of being assigned to the telemedicine group).

Expenses on the part of participants are not expected.

You will be notified promptly if any additional information becomes available during the study that may affect your consent to continue participating in the study.

All information obtained from your medical records and medical history will be treated as confidential. You have the right to access your health information. The results of this study may be published without indicating your identity.

## Appendix E. Addendum to the Informed Consent for a Telemonitoring Group (Translated from Russian)

A purpose of remote health monitoring as part of a study assessing the efficacy of telemedicine technologies in patients with inflammatory bowel diseases:

A procedure for remote monitoring of the patient’s health status, and consultations using telemedicine technologies, are carried out in accordance with the Order of the Ministry of Health of the Russian Federation No 965N dated 30 November 2017 “On approval of the procedure for organizing and providing medical care using telemedicine technologies”.

Remote monitoring of the patient’s health status is aimed at timely detection and prevention of complications, exacerbations of diseases, increasing adherence to treatment and control, prevention, and developing skills to preserve and maintain health.

A clinical goal is to reduce a frequency of relapses in inflammatory bowel disease.

Program:

- Treatment regimen for the period of remote monitoring is prescribed by the attending physician upon discharge from the gastroenterological hospital
- Duration of remote monitoring is 6 months
- List of controlled parameters:
  (1) Body weight
  (2) Complete blood count
  (3) C-reactive protein
  (4) Fecal calprotectin
  (5) A total score of the IBD Disk questionnaire for all patients (results are interpreted by researchers)
  (6) Indicators of Simple Clinical Colitis Activity Index (SCCAI) for patients with ulcerative colitis and the Harvey-Bradshaw Index for patients with Crohn’s disease (results are interpreted by researchers)
- Target parameters:
  ⚬ Hemoglobin—reference values 117–160 g/L
  ⚬ Leukocytes—reference values 4–11 × 10^9^/L
  ⚬ CRP—reference values 0–5 mg/L
  ⚬ Fecal calprotectin—reference values up to 200 µg/g for patients enrolled in the trial with the initial normal level; for patients enrolled in the trial with the initial high level (more than 800 µg/g), the reference value is determined individually after 3 and 6 months of observation
  ⚬ IBD-disk score—reference values 0–40
  ⚬ Simple Clinical Colitis Activity Index (SCCAI)—a score of 0–4 during a remission
  ⚬ Harvey-Bradshaw Index—a score of 0–4 during a remission
- Critical levels of deviations in the values of monitored parameters (indicators for a patient and a doctor):
  ⚬ Hemoglobin—lower than 110 g/L, higher than 170 g/L
  ⚬ Leukocytes—lower than 3 × 10^9^/L, higher than 11 × 10^9^/L
  ⚬ CRP—higher than 10 mg/L
  ⚬ Fecal calprotectin—higher than 200 µg/g for patients enrolled in the trial with the initial normal level; maintaining the same values for patients enrolled in the trial with the initial high level (more than 800 µg/g).
- Critical levels of deviations in the values of monitored parameters (indicators only for a doctor):
  ⚬ IBD-disk score—higher than 40
  ⚬ Simple Clinical Colitis Activity Index (SCCAI) for patients with ulcerative colitis—a score is higher than 5
  ⚬ Harvey-Bradshaw Index for patients with Crohn’s disease—a score is higher than 5

Procedure:

Monitored parameters should be measured and entered into the patient’s personal account with the following regularity:

(1) Complete blood count—once a month
(2) Fecal calprotectin, C-reactive protein—once every 3 month
(3) A total score of the IBD-disk questionnaire—once a month for all patients
(4) Simple Clinical Colitis Activity Index (SCCAI) for patients with ulcerative colitis and the Harvey-Bradshaw Index for patients with Crohn’s disease—once a month.

A follow-up visit with a specialist is expected after 6 months of remote monitoring in the absence of critical deviations and situations requiring emergency medical care.

To provide telemonitoring, patients need to have access to electronic communications and the internet.

Consultations using telemedicine technologies are carried out on a planned basis or at the patient’s request by exchanging messages from Monday to Friday from 9:00 to 17:30 in conditions that are not accompanied by a threat to the patient’s life, do not require emergency and urgent medical care, if a delay in medical care for a certain period of time does not entail a worsening in the patient’s condition, and if it is not a threat to his/her life and health.

A research physician conducting remote monitoring of the patient’s health status reacts immediately if health indicators deviate from the limit values, or complaints indicate the development of an acute condition occurring during the period from Monday through Friday from 9:00 to 17:30. This emergency response covers a communication with the patient to clarify the condition and exclude unreasonable anxiety, informing an attending physician about the situation and measures taken, a supervision of the patient’s call of an emergency ambulance for the hospitalization, or a communication with the attending physician about the emergency hospitalization.

In the case of conditions requiring an emergency response outside the above-mentioned time (Monday—Friday from 9:00 to 17:30), patients should call an emergency ambulance on their own and also inform an attending physician of the hospital about it.

List of conditions requiring emergency response (calling an emergency ambulance):

- Acute abdominal pain, not relieved by taking antispasmodics, lasting more than 30 min, for women—unrelated to menstruation
- Gastrointestinal bleeding
- Fever above 38.5 °С for 5 or more days in the absence of catarrhal symptoms (runny nose, cough, sore throat)
- Signs of intestinal obstruction—cramping abdominal pain, retention of stools and gases, bloating and asymmetrical abdomen, nausea and vomiting
- Signs of perforation of a hollow organ—severe diffuse abdominal pain, nausea, vomiting, moderate palpitations, decreased blood pressure

List of conditions requiring urgent response (communication with an attending physician of the hospital):

- Exacerbation of inflammatory bowel disease (increased stool frequency, abdominal pain (not meeting the emergency response criteria), blood in the stool)
- A fistula of the anterior abdominal wall, perianal, enterovesical, colorectal-vaginal (according to the results of self-examination or examination by a specialist)

Exacerbation of other chronic conditions, as well as the occurrence of emergency situations not related to IBD, require seeking emergency medical care outside of the ongoing study.

## References

[1] Burisch J., Jess T., Martinato M., Lakatos P.L. (2013). The Burden of Inflammatory Bowel Disease in Europe. J. Crohns Colitis.

[2] Coward S., Clement F., Benchimol E.I., Bernstein C.N., Avina-Zubieta J.A., Bitton A., Carroll M.W., Hazlewood G., Jacobson K., Jelinski S. (2019). Past and Future Burden of Inflammatory Bowel Diseases Based on Modeling of Population-Based Data. Gastroenterology.

[3] Singh S., Qian A.S., Nguyen N.H., Ho S.K.M., Luo J., Jairath V., Sandborn W.J., Ma C. (2022). Trends in U.S. Health Care Spending on Inflammatory Bowel Diseases, 1996–2016. Inflamm. Bowel Dis..

[4] Lönnfors S., Vermeire S., Greco M., Hommes D., Bell C., Avedano L. (2014). IBD and Health-Related Quality of Life—Discovering the True Impact. J. Crohns Colitis.

[5] Turner D., Ricciuto A., Lewis A., D’Amico F., Dhaliwal J., Griffiths A.M., Bettenworth D., Sandborn W.J., Sands B.E., Reinisch W. (2021). STRIDE-II: An Update on the Selecting Therapeutic Targets in Inflammatory Bowel Disease (STRIDE) Initiative of the International Organization for the Study of IBD (IOIBD): Determining Therapeutic Goals for Treat-to-Target Strategies in IBD. Gastroenterology.

[6] Gravina A.G., Pellegrino R., Durante T., Palladino G., D’Onofrio R., Mammone S., Arboretto G., Auletta S., Imperio G., Ventura A. (2023). Telemedicine in Inflammatory Bowel Diseases: A New Brick in the Medicine of the Future?. World J. Methodol..

[7] Gravina A.G., Pellegrino R., Cipullo M., Palladino G., Imperio G., Ventura A., Auletta S., Ciamarra P., Federico A. (2024). May ChatGPT Be a Tool Producing Medical Information for Common Inflammatory Bowel Disease Patients’ Questions? An Evidence-Controlled Analysis. World J. Gastroenterol..

[8] Stafford I.S., Gosink M.M., Mossotto E., Ennis S., Hauben M. (2022). A Systematic Review of Artificial Intelligence and Machine Learning Applications to Inflammatory Bowel Disease, with Practical Guidelines for Interpretation. Inflamm. Bowel Dis..

[9] Keil R., Wasserbauer M., Zádorová Z., Kojecký V., Hlava Š., Št’ovíček J., Chudý J., Roznětinská M., Drábek J., Kubišová N. (2018). Adherence, Risk Factors of Non-Adherence and Patient’s Preferred Treatment Strategy of Mesalazine in Ulcerative Colitis: Multicentric Observational Study. Scand. J. Gastroenterol..

[10] Al Khoury A., Balram B., Bessissow T., Afif W., Gonczi L., Abreu M., Lakatos P.L. (2022). Patient Perspectives and Expectations in Inflammatory Bowel Disease: A Systematic Review. Dig. Dis. Sci..

[11] Pang L., Liu H., Liu Z., Tan J., Zhou L., Qiu Y., Lin X., He J., Li X., Lin S. (2022). Role of Telemedicine in Inflammatory Bowel Disease: Systematic Review and Meta-Analysis of Randomized Controlled Trials. J. Med. Internet Res..

[12] Cross R.K., Langenberg P., Regueiro M., Schwartz D.A., Tracy J.K., Collins J.F., Katz J., Ghazi L., Patil S.A., Quezada S.M. (2019). A Randomized Controlled Trial of TELEmedicine for Patients with Inflammatory Bowel Disease (TELE-IBD). Am. J. Gastroenterol..

[13] Shumskaya Y.F., Akhmedzyanova D.A., Mnatsakanyan M.G., Kolosova K.Y., Tashchyаn O.V., Yurazh M.V., Reshetnikov R.V. (2023). Delphi Method to Determine a List of Questionnaire-Assessed Parameters in the Follow-up of Patients with Inflammatory Bowel Disease. Digit. Diagn..

[14] Chan A.-W., Tetzlaff J.M., Gotzsche P.C., Altman D.G., Mann H., Berlin J.A., Dickersin K., Hrobjartsson A., Schulz K.F., Parulekar W.R. (2013). SPIRIT 2013 Explanation and Elaboration: Guidance for Protocols of Clinical Trials. BMJ.

[15] Walmsley R.S., Ayres R.C., Pounder R.E., Allan R.N. (1998). A Simple Clinical Colitis Activity Index. Gut.

[16] Harvey R.F., Bradshaw J.M. (1980). A Simple Index of Crohn’s-Disease Activity. Lancet.

[17] Irvine E.J., Zhou Q., Thompson A.K. (1996). The Short Inflammatory Bowel Disease Questionnaire: A Quality of Life Instrument for Community Physicians Managing Inflammatory Bowel Disease. CCRPT Investigators. Canadian Crohn’s Relapse Prevention Trial. Am. J. Gastroenterol..

[18] The Whoqol Group (1998). Development of the World Health Organization WHOQOL-BREF Quality of Life Assessment. Psychol. Med..

[19] Meng X., Li S., Shen W., Li D., Lv Q., Wang X., Wang Y., Zang X., Zhang Q., Wang L. (2023). Exploration of the Psychometric Properties of the Novel General Medication Adherence Scale (GMAS) for Chronic Illness Patients. Curr. Med. Res. Opin..

[20] Thayaparan A.J., Mahdi E. (2013). The Patient Satisfaction Questionnaire Short Form (PSQ-18) as an Adaptable, Reliable, and Validated Tool for Use in Various Settings. Med. Educ. Online.

[21] Wu Y., Levis B., Daray F.M., Ioannidis J.P.A., Patten S.B., Cuijpers P., Ziegelstein R.C., Gilbody S., Fischer F.H., Fan S. (2023). Comparison of the Accuracy of the 7-Item HADS Depression Subscale and 14-Item Total HADS for Screening for Major Depression: A Systematic Review and Individual Participant Data Meta-Analysis. Psychol. Assess..

[22] Trieschmann K., Chang L., Park S., Naliboff B., Joshi S., Labus J.S., Sauk J.S., Limketkai B.N., Mayer E.A. (2022). The Visceral Sensitivity Index: A Novel Tool for Measuring GI-symptom-specific Anxiety in Inflammatory Bowel Disease. Neurogastroenterol. Motil..

[23] Bagby M., Taylor G.J., Ryan D. (1986). Toronto Alexithymia Scale: Relationship with Personality and Psychopathology Measures. Psychother. Psychosom..

[24] Shelygin Y.A., Ivashkin V.T., Belousova E.A., Reshetov I.V., Maev I.V., Achkasov S.I., Abdulganieva D.I., Alekseeva O.A., Bakulin I.G., Barysheva O.Y. (2023). Ulcerative Colitis (K51), Adults. Koloproktologia.

[25] Shelygin Y.A., Ivashkin V.T., Achkasov S.I., Reshetov I.V., Maev I.V., Belousova E.A., Vardanyan A.V., Nanaeva B.A., Adamyan L.V., Drapkina O.M. (2023). Clinical Guidelines: Crohn’s Disease (К50), Adults. Koloproktologia.

[26] Naqvi A.A., Mahmoud M.A., AlShayban D.M., Alharbi F.A., Alolayan S.O., Althagfan S., Iqbal M.S., Farooqui M., Ishaqui A.A., Elrggal M.E. (2020). Translation and Validation of the Arabic Version of the General Medication Adherence Scale (GMAS) in Saudi Patients with Chronic Illnesses. Saudi Pharm. J..

[27] Sun D., Chi L., Liu J., Liang J., Guo S., Li S. (2021). Psychometric Validation of the Chinese Version of the Short Inflammatory Bowel Disease Questionnaire and Evaluation of Its Measurement Invariance across Sex. Health Qual. Life Outcomes.

[28] Jowett S.L., Seal C.J., Barton J.R., Welfare M.R. (2001). The Short Inflammatory Bowel Disease Questionnaire Is Reliable and Responsive to Clinically Important Change in Ulcerative Colitis. Am. J. Gastroenterol..

[29] Knowles S.R., Graff L.A., Wilding H., Hewitt C., Keefer L., Mikocka-Walus A. (2018). Quality of Life in Inflammatory Bowel Disease: A Systematic Review and Meta-Analyses—Part I. Inflamm. Bowel Dis..

[30] Simren M., Axelsson J., Gillberg R., Abrahamsson H., Svedlund J., Bjornsson E.S. (2002). Quality of Life in Inflammatory Bowel Disease in Remission: The Impact of IBS-like Symptoms and Associated Psychological Factors. Am. J. Gastroenterol..

[31] Nguyen N.H., Martinez I., Atreja A., Sitapati A.M., Sandborn W.J., Ohno-Machado L., Singh S. (2021). Digital Health Technologies for Remote Monitoring and Management of Inflammatory Bowel Disease: A Systematic Review. Am. J. Gastroenterol..

[32] Milat A., Lee K., Conte K., Grunseit A., Wolfenden L., van Nassau F., Orr N., Sreeram P., Bauman A. (2020). Intervention Scalability Assessment Tool: A Decision Support Tool for Health Policy Makers and Implementers. Health Res. Policy Syst..

